# Identification of specificity determining residues in peptide recognition domains using an information theoretic approach applied to large-scale binding maps

**DOI:** 10.1186/1741-7007-9-53

**Published:** 2011-08-11

**Authors:** Kevin Y Yip, Lukas Utz, Simon Sitwell, Xihao Hu, Sachdev S Sidhu, Benjamin E Turk, Mark Gerstein, Philip M Kim

**Affiliations:** 1 Department of Molecular Biophysics and Biochemistry, Yale University, New Haven, CT, USA; 2 Department of Computer Science and Engineering, The Chinese University of Hong Kong, Hong Kong; 3 Terrence Donnelly Centre for Cellular and Biomolecular Research, University of Toronto, Toronto, Ontario, Canada; 4 Department of Pharmacology, Yale University, New Haven, CT, USA; 5 Program in Computational Biology and Bioinformatics, Yale University, New Haven, CT, USA; 6 Department of Computer Science, Yale University, New Haven, CT, USA; 7 Banting and Best Department of Medical Research, University of Toronto, Toronto, Ontario, Canada; 8 Department of Molecular Genetics, University of Toronto, Toronto, Ontario, Canada; 9 Department of Computer Science, University of Toronto, Toronto, Ontario, Canada

## Abstract

**Background:**

Peptide Recognition Domains (PRDs) are commonly found in signaling proteins. They mediate protein-protein interactions by recognizing and binding short motifs in their ligands. Although a great deal is known about PRDs and their interactions, prediction of PRD specificities remains largely an unsolved problem.

**Results:**

We present a novel approach to identifying these Specificity Determining Residues (SDRs). Our algorithm generalizes earlier information theoretic approaches to coevolution analysis, to become applicable to this problem. It leverages the growing wealth of binding data between PRDs and large numbers of random peptides, and searches for PRD residues that exhibit strong evolutionary covariation with some positions of the statistical profiles of bound peptides. The calculations involve only information from sequences, and thus can be applied to PRDs without crystal structures. We applied the approach to PDZ, SH3 and kinase domains, and evaluated the results using both residue proximity in co-crystal structures and verified binding specificity maps from mutagenesis studies.

**Discussion:**

Our predictions were found to be strongly correlated with the physical proximity of residues, demonstrating the ability of our approach to detect physical interactions of the binding partners. Some high-scoring pairs were further confirmed to affect binding specificity using previous experimental results. Combining the covariation results also allowed us to predict binding profiles with higher reliability than two other methods that do not explicitly take residue covariation into account.

**Conclusions:**

The general applicability of our approach to the three different domain families demonstrated in this paper suggests its potential in predicting binding targets and assisting the exploration of binding mechanisms.

## 1 Background

### 1.1 Protein recognition domains (PRDs) and specificity determining residues (SDRs)

Peptide recognition domains are key elements on proteins with many roles in central signaling pathways [1]. PRDs are involved in many diseases and are a focus of drug discovery efforts [2]. Some viruses co-opt host PRDs via mimicry, emphasizing their relevance to human diseases [3-5]. Representative PRDs include PDZ, SH3 and kinase domains. These domains recognize short (usually around seven to ten amino acids long) motifs in their target proteins. Such motifs often lie in unstructured regions [6] and hence resemble peptides in their conformational flexibility. In particular, PDZ domains recognize hydrophobic C-terminal tails, SH3 domains recognize proline-rich motifs, and kinase domains bind several different classes of motifs; for instance, basophilic kinases bind arginine-rich motifs. As many PRDs are well behaved in solution, they are ideal model systems for studying protein-protein interactions and ligand binding specificity. New approaches, namely phage display [7], protein or peptide arrays [8-10] and oriented peptide libraries [11] have greatly facilitated the measurement of specificities and produced an hitherto unimaginable wealth of binding data. Despite these advances, the residues within the domains that determine their binding specificity have only partly been elucidated. Such specificity determining residues (SDRs) [12,13] can be identified using dedicated experiments involving site-directed mutagenesis and subsequent measurement of specificity. For example, recent studies have directly tested effects of point mutations in kinases [14] and PDZ domains [7]. However, because of the size of the sequence space to be covered, exhaustive experimental search is infeasible. While co-crystal structures of PRDs with the bound ligand are often used to prioritize residues, identification of SDRs remains a complex problem: on the one hand, close proximity to the ligand does not necessarily implicate a residue in specificity determination. On the other hand, a residue that is far away from the ligand can also affect specificity due to secondary effects on the binding site [13,15]. Hence, computational methods are needed to select and prioritize the positions to be tested through mutagenesis. Meanwhile, the aforementioned wealth of specificity data offers ample resources to be computationally analyzed for information about SDRs.

Currently, there are two major classes of computational approaches for SDR identification: first, there are structure driven approaches, making use of physical properties from protein structures, such as the hydration site free energies displaced by ligand atoms upon binding [16]. The second class of approaches adopts a statistical approach, and identifies SDRs by looking for sites that are conserved across or within functional groups [17], or more conserved in orthologs than paralogs [12]. Our method also takes on a statistical approach, the systematic nature of which enables the potential identification of non-local interactions between residues that are significant for peptide binding. Due to the lack of large-scale binding data previously, most current statistical methods attempt to detect SDRs based only on conservation signals from multiple sequence alignments of the PRDs. These predictions are noisy as the conservation of a residue could be due to roles other than binding specificity determination. Hence, these approaches do not make optimal use of currently available data. Conversely, utilizing the information about the actual bound peptide profiles from the recent large-scale binding datasets has the potential to boost the power of new predictions. In this paper we demonstrate how these new datasets can be incorporated in the search for SDRs. Previously we explored this idea using a proof-of-principle correlation-based method as part of a larger experimental study [14]. Here, we develop a new approach that is based on a novel formalism of mutual information.

### 1.2 Traditional and generalized covariation methods

Our approach is based on the notion that SDRs would covary with the binding specificity. The concept of covariation has first been used to study internal residue contacts in proteins [18,19]. It has subsequently been used in the identification of energetically coupled residues [20], coevolution of proteins with their interaction partners [21-25], protein fold [26], functionally or structurally coupled residues within a protein [27,28] and protein fusion [29]. The basic idea is to look for two sites in a multiple sequence alignment (MSA), or a pair of MSAs when studying the interaction between two proteins, that exhibit coordinated changes. Such covariation could suggest functional or structural dependency between the two sites, as there exists evolutionary pressure against their independent evolution. In the context of SDR identification, the covariation between a residue in the PRD and a residue in the bound peptide could imply its role in mediating the binding.

**Figure 1:**
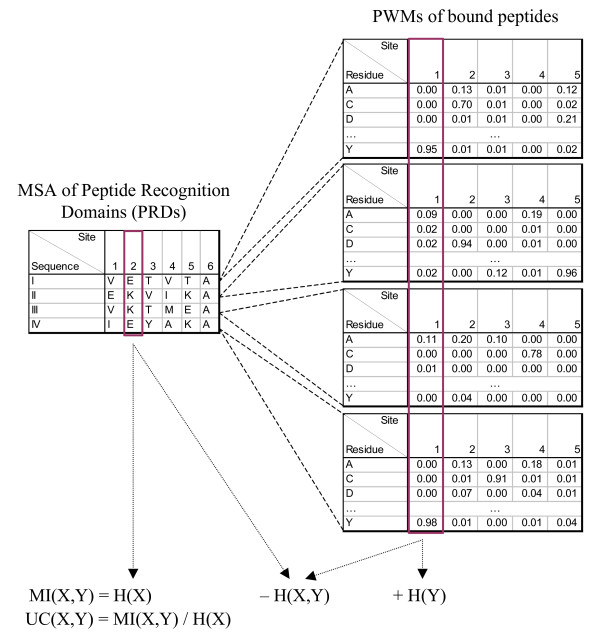
**An illustration of our method for finding specificity determining residues (SDRs)**. The sequences of the peptide recognition domains (PRDs) are aligned to form a multiple sequence alignment (MSA). Each PRD has an associated position weight matrix (PWM) of the peptides that it binds, and the PWMs are also aligned. The entropy of each MSA site *X *and each PWM site *Y *is computed and combined to give the mutual information (MI) and uncertainty coefficient (UC) scores.

Many computational methods have been proposed for identifying covarying sites, including correlation scores [18,30], Statistical Coupling Analysis (SCA) [20,31], likelihood methods [32,33], information theoretic methods [27,34], independence tests [35,36] and Bayesian approaches [37]. However, these methods cannot be applied directly to the identification of SDRs, because in a typical protein-peptide binding dataset [13,38], each PRD does not bind to only one single peptide sequence. Instead, a PRD is associated with a binding profile, often represented in the form of a position weight matrix (PWM). In other words, instead of having two MSAs as in a typical covariation study, SDR identification adopts a more general setting with one MSA of PRDs on the one hand, and a list of respective PWMs on the other hand (Figure 1). Furthermore, a multitude of techniques have been developed in recent studies to handle various issues in the application of the covariation methods [34,39-42], such as uneven representation of sequences in the MSA and overly diverse sites. These techniques also need to be extended in order to be applied in the current more generalized setting. In this paper we present a novel method to determine SDRs using an information theoretic approach. For each site from the MSA of the PRDs and each site from the aligned peptide PWMs, the method produces a numeric covariation score, which can be used as an indicator of the likelihood that the two sites are involved in binding specificity. Aggregating the scores could also suggest which peptides a PRD is likely to bind. Our approach uses only information from sequences, and thus can be applied to PRDs without known crystal structures.

## 2 Methods

### 2.1 Entropy and mutual information

Our method for identifying SDRs is based on an information theoretic measure called mutual information, which has been used in detecting covarying residues in the traditional setting [27,34]. We first give some general definitions here, and then discuss how they are used in the identification of SDRs in the next subsection. Given a discrete random variable *X *with a distribution *p*(*x*) over the domain of *X*, , the entropy of *X *is defined as:(1)

where log 0 is defined as 0, the asymptotic value of log(*x*) towards 0. In the general definition of entropy, the base of the logarithm can be set to any value. In analyzing protein sequences, where  is the set of 20 amino acid residues, it is convenient to use 20 as the base so that the entropy value is always between zero and one [43].

Similarly, if we have two random variables *X *and *Y *with a joint distribution *p*(*x*, *y*), the joint entropy of them is defined as:(2)

Based on the concepts of entropy and joint entropy, the mutual information between two random variables *X *and *Y *is defined as follows:(3)

### 2.2 Adapting mutual information to the identification of SDRs

Suppose we are given an MSA *A *with *n *rows and *m *columns. Each row corresponds to a PRD sequence and each column corresponds to a site of the alignment. We use *A_ij _*to denote the residue at site *j *of sequence *i*. The residues at a site can be viewed as a sample drawn from a distribution of residues specific to the site. Let *A_j _*be a random variable for the residues at site *j*. Using Formula 1, we can calculate the entropy of *A_j _*by replacing *p*(*x*) with the sample distribution, *p_j _*(*x*), defined by the frequency of each residue at the site:(4)

where  is the indicator function with  and .

The entropy can be interpreted as the uncertainty of which residue we would encounter at the site if we randomly draw a sequence from the MSA, where uncertainty here is mathematically quantified by the number of bits needed to encode the information on average. A completely conserved site has an entropy of zero, and indeed there is no uncertainty as the residue being drawn must always be the same. A site with equal probability of all 20 residues has the maximum possible entropy of one. In general, a more diverse site has a larger entropy.

Similarly, suppose we are given a set of *n *aligned PWMs *W *each with *w *sites. The *i*th PWM represents the peptides bound by the *i*th PRD in the MSA (Figure 1). Denote *W_ik _*(*y*) as the probability of residue *y *at the *k*th site of the *i*th PWM. Let *W_k _*be a random variable for the residues at site *k*. Again, we can calculate the entropy of *W_k _*using Formula 1 by replacing *p*(*x*) with the expected probability of *y *in the different PWMs, *p_k _*(*y*), assuming a uniform distribution of the observed sequences:(5)

Now, we can compute the joint entropy of site *j *in the MSA and site *k *in the PWMs using Formula 2, based on the sample distribution of *A_j _*and the probabilities *W_ik _*(*y*):(6)

The joint entropy measures the uncertainty of which two residues we would encounter at MSA site *j *and PWM site *k *if we randomly draw a sequence from the MSA and get its corresponding PWM.

Finally, we can compute the mutual information between MSA site *j *and PWM site *k *using Formula 3. Since *H *(*X*, *Y*) is the uncertainty of the two sites that persists even when we consider them together, subtracting it from *H *(*X*) + *H *(*Y*) gives the uncertainty that is eliminated by considering the two sites together. In other words, mutual information measures the information shared by the two sites. A larger mutual information indicates a stronger dependency between them. This could indicate a functional or structural relationship between the two sites. For example, it could suggest that the two sites coevolve in the sense that when the residue at the MSA site is changed, binding strength is restored by having a corresponding change at the PWM site.

### 2.3 Handling uneven sequence representation

In many cases, the input MSA for studying residue covariation has an uneven representation of sequences from different clades. For example, it is common to have more sequences from model organisms or species that are better studied, and fewer sequences from other species. As a result, the MSA could contain many sequences that are highly similar, and few that are significantly different. Each of the highly similar sequences contributes little additional information, but still has an equal amount of influence on the calculation of mutual information under the assumption of a uniform distribution of the observed sequences. Consequently, they could mask the novel information from low abundance sequences. To counteract this effect, we associate weights with the sequences so that the more unique ones receive higher weights. Statistically, it is equivalent to placing a prior distribution to the observed sequences if the weights are normalized to take values between zero and one and have a sum of one. Here we assume there is an external procedure for determining the weights. For instance, one possible way is to construct a phylogenetic tree from the sequences in the MSA, and then recursively distribute the total weight to different branches of the tree, so that each sequence in the crowded branches will receive a smaller share of weights [44]. Suppose the procedure assigns *α_i _*as the weight of sequence *i*, we can redefine *p_j _*(*x*), *p_k _*(*y*) and *p*_*j*,*k *_(*x*, *y*) as follows:(7)(8)(9)

Entropy, joint-entropy and mutual information can then be calculated using these new definitions of probabilities.

### 2.4 Handling uneven site conservation

A potential issue of the mutual information measure is that a pair of unrelated sites could have even higher mutual information than a pair of truly covarying sites if the unrelated sites are individually much more diverse than the covarying sites. This is illustrated by the hypothetical example shown in Table 1. For simplicity, suppose the sequences all have equal weights and the base of logarithms is two. Sites 1 and 2 are truly covarying. When site 1 changes from the non-polar residue alanine in sequences I and II to the polar residue threonine in sequences III and IV, site 2 simultaneously changes from the non-polar residue valine to the polar residue tyrosine. The entropy of each of the two sites is one and the mutual information between them is also one, the maximum possible value given the two individual entropy values. On the other hand, sites 3 and 4 are random, unrelated sites. The entropy values of them are 2 and 1.5, respectively, and their mutual information is 1.5, which is higher than the mutual information between sites 1 and 2 due to larger entropy values of sites 3 and 4.

**Table 1:** A hypothetical example MSA that illustrates the problem of uneven site conservation.

Sequence/Site	1	2	3	4
I	A	V	A	C
II	A	V	C	D
III	T	Y	D	E
IV	T	Y	E	C

Sites 1 and 2 are truly covarying, while sites 3 and 4 are random and unrelated. However, as sites 3 and 4 have higher entropy values than sites 1 and 2, the resulting mutual information between sites 3 and 4 is also higher than that between sites 1 and 2 (see main text for the calculations).

To deal with this problem, various kinds of normalization have been proposed to penalize overly diverse sites [34]. We will focus on the uncertainty coefficient [45], which was found to be one of the best normalized mutual information scores in our preliminary study. For an MSA site *A_j _*and a PWM site *W_k_*, the uncertainty coefficient is defined as follows:(10)

We have also tried handling the problem using a statistical test. Specifically, we used mutual information as the test statistic to calculate how unlikely it is to get a mutual information at least as large as the observed one under the null hypothesis that the two sites are independent. The distribution of mutual information can be obtained by permuting the residues of a site, or by using a chi-square approximation. It was shown that when *n *is large, (2 ln 2*n*)*MI*(*X*, *Y*) tends to have a chi-square distribution with  degrees of freedom [46,47]. It turns out that the results based on this statistical test were not better than using the simple normalization approach. We thus focus on the use of the uncertainty coefficient measure in handling uneven site conservation in the remaining of this paper.

Table 1 also demonstrates the tradeoff between the mutual information between two sites and their individual conservation, both of which are indicators of their functional importance. One may try to derive a measure that takes both into account, similar to what the Sequence Harmony method handles both the conservation and similarity of two groups of residues simultaneously, for the problem of identifying important residues that determine the functional differences of protein subfamilies [48]. We leave the derivation of a new covariation measure to a future study.

### 2.5 Predicting the PWMs of bound peptides

One important use of the covariation scores is to contribute towards predicting the PWMs of bound peptides. This can be done by aggregating the detected covariation signals. Suppose we are given a new PRD sequence (the (*n *+ 1)th sequence) of the MSA *M *without the corresponding PWM of its bound peptides. We would like to predict the PWM based on the *n *+ 1 sequences in the MSA and the *n *known PWMs. We investigate the use of the covariation scores in this problem by comparing a prediction method that considers site covariation with two methods that do not.

A simple prediction method that does not take site covariation into account is to perform a weighted averaged of the known PWMs, where the weights are based on the similarity of the new PRD sequence and the original ones. Specifically, the probability of finding residue *y *at site *k *of the bound peptides of the new PRD is predicted by the following formula:(11)

where *s*(*i, i'*) is a similarity between sequences *i *and *i' *in the MSA, such as their sequence identity:(12)

Using the covariation scores, we propose an alternative way to define the similarity function. Each MSA site receives a different weight in the calculation, where the weight depends on the covariation score between the site and the target PWM site *k*. In other words, the similarity score *s*(*i, i'*) is replaced with a new score *s_k _*(*i, i'*) that is specific to *k*:(13)(14)

where the uncertainty coefficient *UC*(*A_j_, W_k_*) is computed based on the *n *original sequences.

We also investigated if prediction accuracy can be improved by using a more complex model. Specifically, we treated each MSA site as a categorical attribute and trained a regression tree model for each probability value in the PWM. The models were then applied to the new sequence to predict the PWM of its bound peptides. We implemented this method using REPTree of the Weka package [49].

To evaluate the effectiveness of the different methods, we performed left-out validation as follows. Each time, we drew a random sample of PRDs to form the testing set. Each sequence in the testing set took turn to take the role of the (*n *+ 1)th sequence. The sequences not included in the sample formed the training set. These sequences were used to compute covariation scores and train prediction models. The procedure was repeated 1,000 times for PDZ and SH3 and 50 times for kinase (due to the long running time) with different random training-testing splits, and the average performance of the trained models on the testing sets was recorded. To eliminate near-identical sequences in the training and testing sets, for sequences with 90% or more identity, we kept only one of them in the dataset before making the training-testing splits. As most of the synthetic PDZ sequences (described below) are highly similar, we excluded this dataset from this part of study.

Each predicted PWM was compared to the actual PWM, and a prediction error was computed as the root-mean-square difference between their distributions per site:(15)

where  and  are the predicted and actual PWMs for the bound peptides of the testing sequence, respectively, and the inner summation is taken over all 20 amino acid residues.

## 3 Results

### 3.1 Application of the method to PDZ, SH3 and kinase domains

#### 3.1.1 Natural PDZ domains

We obtained 33 class I human and worm PDZ domains from a recent large-scale study on the specificity map of PDZ domains [13]. Class I PDZ domains were defined by two positions on the ligand, with the pattern *X*[*T/S*]*Xϕ_COOH_*, where *X *and *ϕ *represent a residue and a hydrophobe, respectively. In the same study, a number of SDRs were experimentally determined, allowing us to validate our prediction results. We focused on only one class of domains as the sequences in different classes are difficult to align due to divergence. The pairwise sequence identity ranges from 0.13 to 0.87, with an average of 0.28.

The binding profile of each domain, in the form of a PWM, was obtained from phage display experiments that expressed a random library of C-terminal peptides [13]. The domains were then aligned to form an MSA using ClustalW [50] followed by manual corrections of some obvious errors. A phylogenetic tree of the MSA sequences was constructed using Biopython's Nexus module [51], and the tree was used to produce sequence weights according to a described algorithm [44]. The uncertainty coefficient between each MSA site and each site of the peptide PWMs was computed. To reduce noise and eliminate highly conserved sites that provide little information about covariation, we considered only sites with no gaps [52] and the most frequent residue occupying no more than half of the total sequence weights. This filtering was also applied to the other domain families described below.

The remaining unfiltered site pairs were then evaluated in two ways. First, their uncertainty coefficients were compared to their physical distances in the co-crystal structure 2H2C of ligand-bound human ZO-1 PDZ1 domain [53] in PDB [54]. Although proximity does not necessarily mean functional or structural dependency, it is usually used as a quick check in covariation studies [18,27,29,33]. It also provides a complete and unbiased alternative to the more costly experimental validations.

Second, we examined the top-scoring site pairs, and compared them with known SDRs from a mutagenesis study [13] in which ten sites of the ERBB2IP-1 domain were mutated and the corresponding changes of peptide specificity reported. This comparison provides direct evaluation of our SDR identification procedure on the subset of sites that were tested in the assay.

#### 3.1.2 Synthetic PDZ domains

In a recent study, the mutagenesis study in Tonikian *et al*. [13] was extended. A large amount of mutations were introduced at the ten sites, resulting in 61 variations of the Erbin domain that are functional in recognizing some C-terminal heptapeptides [55]. As with the natural PDZ domains, we compared the uncertainty coefficients with the physical distances in the 2H2C PDB structure. Since the synthetic PDZ domains are 100% conserved at sites other than the ten selected ones, and have a specific set of mutations at the ten sites introduced by the mutagenesis experiments, their MSA exhibits some statistical properties different from those of the natural PDZ domains.

#### 3.1.3 SH3 domains

We obtained 23 yeast SH3 domains and the corresponding PWMs of the bound peptides from phage display experiments from a recent study [38]. We aligned the PRDs based on a published structural alignment [15], and aligned the peptide PWMs based on both the general PxxP pattern and some published alignments [15,56]. The pairwise sequence identity ranges from 0.07 to 0.79, with an average of 0.24.

We applied the same prediction and evaluation procedures as in the case of PDZ, except that in this case we did not have large-scale mutagenesis data, and therefore the prediction results were only evaluated against the physical distances calculated from the crystal structure 1N5Z in PDB, which contains the yeast Pex13 SH3 domain bound to a Pex14 peptide [57], and the findings of some previous studies.

#### 3.1.4 Kinase domains

We also obtained 149 serine/threonine protein kinase domains from four species (*S. cerevisiae*, *H. sapiens*, *S. pombe *and *D. discoideum*) and the PWMs of their corresponding bound peptides [14]. The MSA was made using MUSCLE [58] followed by some manual corrections. The prediction results were evaluated against distances calculated from the crystal structure 1ATP of mouse catalytic subunit of cAMP-dependent protein kinase complexed with Mn-ATP and a peptide inhibitor [59] in PDB, and some findings in previous studies. The pairwise sequence identity ranges from 0.09 to 0.92, with an average of 0.23.

### 3.2 Covariation score correlates with physical proximity and reconfirms previous findings

The covariation score between two sites is found to correlate negatively with the physical distance between them, regardless of the exact definition of the distance measure [see Additional file 1 Figure S1, Additional file 2 Figure S2 and Additional file 3 Figure S3 for the results] when the distance between residue centers, alpha carbon atoms and closest atoms minus their van der Waals' radii were used, respectively. All *P*-values were computed using Fisher transformation [60]. For PDZ, we have also compared the correlations based on several other PDZ structures, and observed similar patterns [Additional file 4 Figure S4].

**Figure 2:**
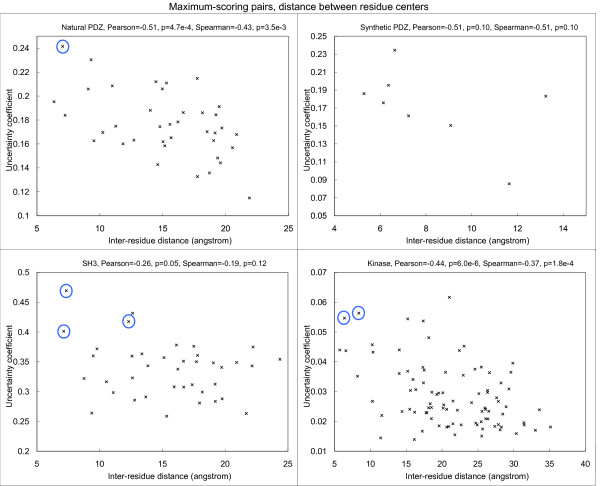
**Correlation between covariation score and physical proximity between each PRD site and its highest-scoring peptide PWM position for the four types of PRDs**. Selected top-scoring pairs discussed in the text are circled. The correlation for synthetic PDZ was not statistically significant due to the small number of data points involved.

Since low-scoring pairs are more subject to noise, here for each PRD site, we focus on the peptide site that gives the highest uncertainty coefficient with it (Figure 2). The site pairs with the highest covariation scores are listed in Table 2.

**Table 2:** Site pairs with the highest covariation scores.

Domain family Ref. PDB Structure rank	Natural 2H2C		Synthetic PDZ 2H2C		SH3 1N5Z		Kinase 1ATP	
	PRD	Peptide	PRD	Peptide	PRD	Peptide	PRD	Peptide
1	Leu60	Trp119	His88	Thr118	Asn71	Leu7	Tyr229	Ile22
2	Val55	Thr117	Ala37	Thr117	Leu34	Leu7	Leu205	Ile22
3	Ala76	Thr117	Ser39	Thr117	Ile73	Leu7	Leu198	Ile22
4	Pro30	Trp119	Leu60	Thr117	Tyr72	Leu7	Lys189	Ile22
5	Ser98	Trp119	Val92	Thr118	Lys35	Leu7	Phe129	Ile22
6	Ala89	Thr117	Ser57	Thr117	Ser44	Leu7	Glu230	Ile22
7	Gln93	Trp119	Asp58	Thr117	Lys61	Leu7	Glu203	Ile22
8	Asp58	Thr117	Phe34	Thr118	Ile68	Leu7	Ile180	Ile22
9	Ala37	Thr117			Leu20	Leu7	Phe187	Arg19
10	Lys103	Trp119			Ala17	Leu7	Cys199	Ile22

For each site on the PRD, only the site on the peptide with the highest score is shown. For synthetic PDZ domains, only ten sites have variations and among them two were filtered, leaving only eight valid sites. All sites are indexed according to their residue numbers in the reference PDB structures.

**Figure 3:**
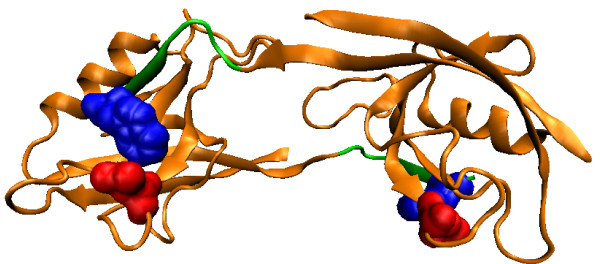
**Top-scoring residue pair for the natural PDZ domain**. The PDZ domain (orange) and the ligand (green) in the biological assembly (dimer) of the PDB structure 2H2C are shown. The top-scoring residue pair between Leu60 (red) on the domain and position -1 of the binding motif on the ligand (blue) are in physical contact.

For natural PDZ domains, the highest-scoring pair (circled) is between Leu60 (*β*3:*α*1-1, structural nomenclature from [53]) of the PDZ domain in 2H2C and position -1 of the binding motif on the bound peptide. These residues are in physical contact in the dimer structure (Figure 3, all visualization produced using VMD [61]). Interestingly, it has been reported that the side chain at *β*3:*α*1-1 can contribute to the recognition of the -1 position of the motif [53], and in the crystal structure from Shank1, a salt bridge is observed between Asp(*β*3:*α*1-1) of the PDZ domain and Arg(-1) of the ligand [62]. Our covariation analysis has thus identified these verified SDRs of the PDZ domains in silico.

**Figure 4:**
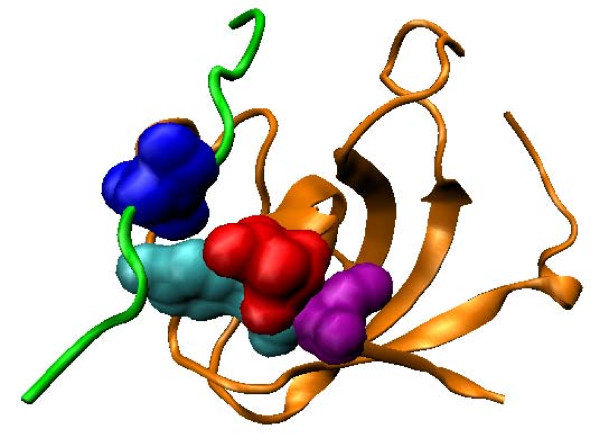
**Top-scoring residue pair for the SH3 domain**. The SH3 domain (orange) and the ligand (green) in the PDB structure 1N5Z are shown. The top-scoring residue pair between Asn71 (red) on the domain and Leu7 of the ligand (blue) is in close physical proximity. Also shown are the MSA residues in the third (Ile73) and fourth (Tyr72) top-scoring pairs.

For SH3 domains, the highest-scoring pair is between Asn71 of the SH3 domain and Leu7 (P+2 residue) of the ligand in 1N5Z. These residues are in close physical proximity in the crystal structure (Figure 4). Interestingly, we found that the MSA residues in the first (Asn71), third (Ile73) and fourth (Tyr72) top-scoring pairs are consecutive in the protein sequence, and two of them are close to Leu7 of the ligand. In a previous study [63], the corresponding residue of Tyr72 on P53BP2, which is an unusual leucine, was hypothesized to cause the protein to bind a peptide very different from its usual ligands. However, mutating the leucine to tyrosine did not affect recognition specificity. As the corresponding residue of Ile73 on P53BP2 is also mutated from the class consensus, and the covariation scores for Asn71 and Ile73 are also high, the three residues may have some combined effects in affecting recognition specificity.

It is also known that the residue corresponding to Glu31 in the RT loop of SH3 domains is a major determinant of the identity of the P-3 residue of the ligand [64,65]. Since the P-3 residue does not exist in the 1N5Z structure, it is not included in the correlation plots. However, when we checked the covariation scores, indeed the ligand residue having the highest score with Glu31 is the P-3 residue. This observation illustrates the potential of our method to identify SDRs when structural information is not available.

**Figure 5:**
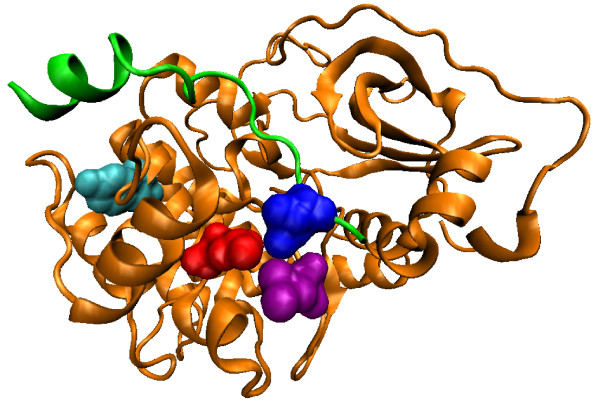
**Top-scoring residue pair for the kinase domain**. The kinase domain (orange) and the ligand (green) in the PDB structure 1ATP are shown. The second top-scoring residue pair between Leu205 (red) on the domain and position 1 of the binding motif on the ligand (blue), as well as the third top-scoring residue pair between Leu198 (purple) and position 1 of the ligand motif, are in close proximity. The MSA residue Tyr229 involved in the top-scoring pair is also shown (cyan).

For protein kinases, the top-scoring pair between Tyr229 of the kinase domain in 1ATP and position +1 of the binding motif in the bound peptide is not physically close. However, the next two pairs (between Leu205 and position +1, and between Leu198 and position +1) are both close in proximity (Figure 5). Both Leu198 and Leu205 were previously found to have hydrophobic interactions with position +1 of the peptide [66,67]. They are two of the three residues (the third being Pro202, which has the highest covariation score with position +1 as compared to other peptide positions) that form the binding pocket for position +1 of the peptide, and contributes to the positioning of a significant portion (-3 to +1 positions) of the bound peptide [67].

We found that the pair between Tyr229 and position +1, besides being the top-scoring pair when uncertainty coefficient was used as the covariation score, was also consistently one of the highest-scoring pairs when covariation was measured by other normalized forms of mutual information. These consistent results made us hypothesize that Tyr229 is also involved in determining binding specificity of the kinase domain, and has long-range coupling with position +1 of the bound peptide. In a previous study, this residue was predicted to be involved in linking nucleotide binding and peptide binding in protein kinases [68]. Interestingly, in the study Tyr229 and Leu205 are predicted to belong to the same special network (termed the *θ*-shaped network) of related residues. It thus might be the case that Tyr229 acts through the residues in the network to affect the recognition of the +1 position of the peptide.

### 3.3 PDZ predictions are consistent with mutagenesis results

We further validated our predictions with the natural PDZ domains by using a mutagenesis dataset from a domain specificity study [13] as described in the Materials and Methods section.

In our covariation calculation, among the ten mutated sites, four were filtered as they were too conserved (Ile36 [*β*2-1], Ile38 [*β*2-3], His88 [*α*2-1] and Val92 [*α*2-5]). Interestingly, in the mutagenesis study, indeed no significant changes to the peptide PWM could be observed for Ile36 and Ile38. The high conservation of them is thus probably caused by a structural or functional role that is independent of peptide binding.

For the remaining six sites, we examined their maximum-scoring peptide residues. Four out of the six had significant changes of the PWM at the predicted positions in the mutagenesis study (Table 3), including the top-scoring pair among all predictions, between Leu60 and position -1. For the remaining two, changes were also observed, albeit with lower statistical significance.

**Table 3:** Validation results of our PDZ predictions.

PDZ site in 2H2C	**Structure-based nomenclature **[53]	Ligand position	Uncertainty coefficient	Distance (Å)	Specificity change
Phe34	*β*1:*β*2-7	-3	0.16	15.0	Significant
Ala37	*β*2-2	-3	0.20	6.4	Significant
Ser39	*β*2-4	-1	0.16	9.5	Significant
Ser57	*β*3-4	-3	0.18	7.2	Observed
Asp58	*β*3-5	-3	0.21	9.1	Observed
Leu60	*β*3:*α*1-1	-1	0.24	7.0	Significant

The six sites validated in Tonikian *et al*. [13] that were not filtered in our calculation are shown, along with their highest-scoring peptide PWM positions. Significant changes of the predicted PWM positions after mutating the PDZ sites were reported for four cases, while in the remaining two cases some changes were also observed.

The predicted pair between Phe34 and position -3 has a distance of 15.0 Å in the PDB structure 2H2C. If the SDRs of different class I PDZ domains are similar, this predicted pair is another example that suggests our covariation method could potentially identify physically distant SDR pairs.

### 3.4 Covariation scores improve prediction of bound peptide profiles

**Figure 6:**
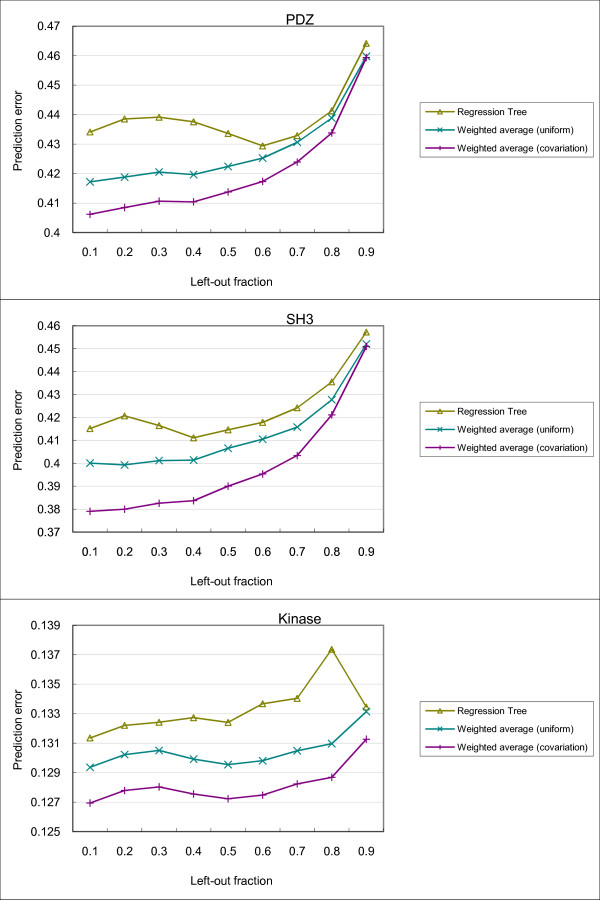
**Results of predicting PWMs of bound peptides**. Three methods for predicting PWMs of bound peptides were compared using data from (top) natural PDZ domains, (middle) SH3 domains and (bottom) kinase domains.

As described in the Materials and Methods section, we compared three different methods for predicting the profiles of bound peptides. The prediction results are shown in Figure 6.

In general, the prediction error is lower with a smaller fraction of PRDs left out for testing. This is expected, as having more PRDs in the training set allows for more accurate computation of covariation scores and the construction of more informative prediction models.

In all cases, the weighted average method using covariation scores outperformed regression tree and the weighted average method using uniform scoring. This suggests that the covariation score provided a meaningful way to weight useful features (that is, PRD sites) for predicting residue distributions of the bound peptides. Interestingly, while the regression tree method also performed feature weighting, in general it performed worse than both weighted average methods. The low performance could be due to over-fitting, as the regression trees are rich in expressive power while the numbers of PRDs in the datasets are small.

## 4 Discussion

We present a novel way to use underutilized data. Our method is a valuable tool for exploring the specificity space of PRDs. Moreover, as the amount of specificity data is increasing swiftly, also due to the advent of next-generation sequencing and its applications to phage display [69], our method will prove even more valuable to make optimal use of this kind of data.

We think our covariation approach can be used in conjunction with other methods to improve SDR predictions. Since most current approaches are based on force fields and structural methods, our method opens up a new perspective for improvement. As shown in the recent PRD specificity prediction challenge of the DREAM4 competition [70], most likely a combination of structural and statistical methods will be most successful at predicting specificities.

One limitation of our approach is that it does not consider possible relationships between different residue pairs. As multiple PRD residues could simultaneously interact with a peptide residue and multiple peptide residues could simultaneously interact with a PRD residue [71], binding specificity could be more accurately modeled by considering covariation signals between residue groups. Furthermore, since a residue could have an indirect covariation signal with another residue through an intermediate residue [42], performing residue group analysis could help filter out these non-SDR intermediates that have relatively high covariation signals.

Another limitation of our approach is that it fails to identity SDRs that are highly conserved. Indeed we have observed that in PDZ, some highly conserved residues (for example *α*2-1) are physically close to the peptide and have been experimentally shown to affect binding specificity when mutated [13] [Additional file 5 Figure S5]. On the other hand, some SDRs are not highly conserved, but exhibit strong covariation patterns with peptide residues (for example *β*3:*α*1-1). Future approaches could improve upon our current method by combining information about conservation with covariation to identity SDRs.

There are also PRD residues that determine binding but not binding specificity in that if they are mutated, the resulting effect on binding cannot be restored by a second mutation. For instance, if a residue is critical to the protein structure, mutating it could seriously affect the stability of the protein, which in turn affects peptide binding. These residues are likely to be very conserved, and thus would not be ranked high by our method.

A third limitation of our approach, and more generally of all covariation analysis methods based on a multiple sequence alignment, is the dependency on the alignment quality. Different alignments could give very different results, especially for alignments with many gaps. To cope with this issue, we have used a published alignment for SH3, and made some manual corrections to the PDZ and kinase alignments generated by computer programs. Future approaches should try to minimize the effect of alignment quality on the analysis results.

While we have attempted to predict peptide PWMs, it is also possible to predict interactions given a PRD and a peptide. This problem has recently been studied using some large-scale datasets [9,52]. It would be interesting to study how the concept of covariation can be incorporated into these prediction methods.

## Conclusions

We have presented a novel approach that utilizes an as of yet underused source of data. We have shown that the covariation scores are consistent with previous findings from both a large-scale study, and other individual experiments. In addition, we have identified a number of candidate SDRs in a ranked list for future experimental validation. In particular, with the top-scoring pairs from natural PDZ domains and kinase domains both verified in previous work, the SH3 top-scoring pairs are good candidates for testing their roles in determining the binding specificity of SH3 domains.

## Abbreviations

MI: mutual information; MSA: multiple sequence alignment; PRDs: peptide recognition domains; PWM: position weight matrix; SCA: statistical coupling analysis; SDRs: specificity determining residues; UC: uncertainty coefficient.

## Competing interests

The authors declare that they have no competing interests.

## Authors' contributions

All authors conceived the project and design. KY, LU, SS and PK prepared the data. KY, LU, SS and XH implemented the algorithms, performed the computational experiments, and analyzed the results. KY, LU, MG and PK wrote the paper. All authors read and approved the document.

## Supplementary Material

Additional file 1 **Correlation between covariation score and physical proximity between each PRD site and each PWM position for the three types of PRDs when distances are computed between residue centers**. Figure S1. Click here for file

Additional file 2 **Correlation between covariation score and physical proximity between each PRD site and each PWM position for the three types of PRDs when distances are computed between alpha carbon atoms**. Figure S2. Click here for file

Additional file 3 **Correlation between covariation score and physical proximity between each PRD site and each PWM position for the three types of PRDs when distances are computed between the closest atoms minus their van der Waal's radii**. Figure S3 Click here for file

Additional file 4 **Correlation between covariation score and physical proximity between each PRD site of a PDZ domain and each PWM position when distances are computed between alpha carbon atoms in four different PDB structures**. Figure S4 Click here for file

Additional file 5 **Conservation and distance to the closest peptide residue of each PDZ domain site. For each site on the domain, we computed the total sequence weight of the sequences having a particular amino acid at the site. The conservation of the site is defined by the maximum of such total weights normalized by the total sequence weight of all sequences in the MSA**. Figure S5. Click here for file
