# Supplementary material for: Identification of specificity determining residues in peptide recognition domains using an information theoretic approach applied to large-scale binding maps
Source: BMC Biol. 2011 Aug 11;9:53. doi: 10.1186/1741-7007-9-53 (PMC3224579; doi:10.1186/1741-7007-9-53)
Supplement: Additional file 1 — Correlation between covariation score and physical proximity between each PRD site and each PWM position for the three types of PRDs when distances are computed between residue centers. Figure S1. [file 1741-7007-9-53-S1.PDF]

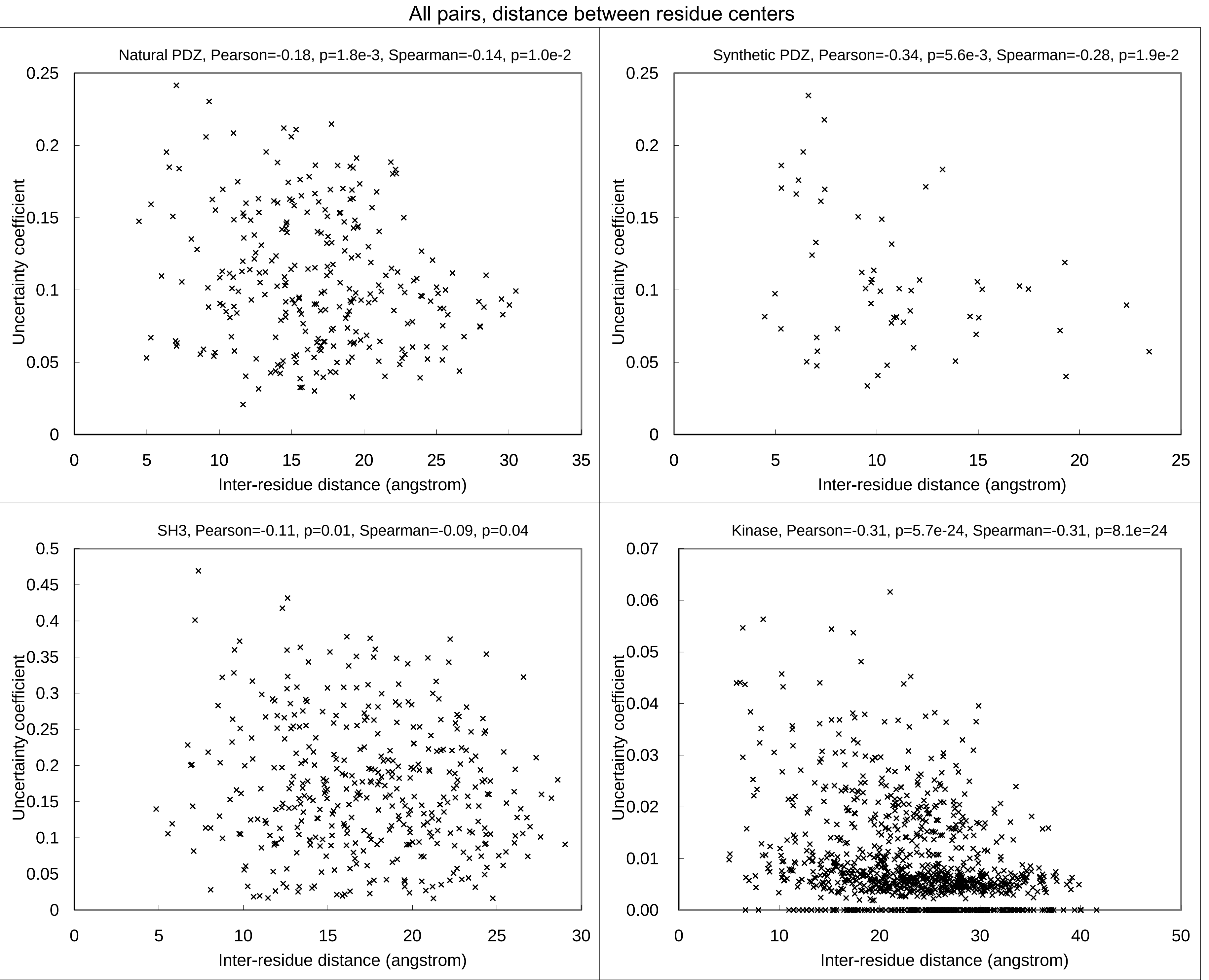
All pairs, distance between residue centers
Natural PDZ, Pearson=-0.18, p=1.8e-3, Spearman=-0.14, p=1.0e-2
Synthetic PDZ, Pearson=-0.34, p=5.6e-3, Spearman=-0.28, p=1.9e-2
SH3, Pearson=-0.11, p=0.01, Spearman=-0.09, p=0.04
Kinase, Pearson=-0.31, p=5.7e-24, Spearman=-0.31, p=8.1e=24
Uncertainty coefficient
Inter-residue distance (angstrom)
