# Supplementary material for: Identification of specificity determining residues in peptide recognition domains using an information theoretic approach applied to large-scale binding maps
Source: BMC Biol. 2011 Aug 11;9:53. doi: 10.1186/1741-7007-9-53 (PMC3224579; doi:10.1186/1741-7007-9-53)
Supplement: Additional file 2 — Correlation between covariation score and physical proximity between each PRD site and each PWM position for the three types of PRDs when distances are computed between alpha carbon atoms. Figure S2. [file 1741-7007-9-53-S2.PDF]

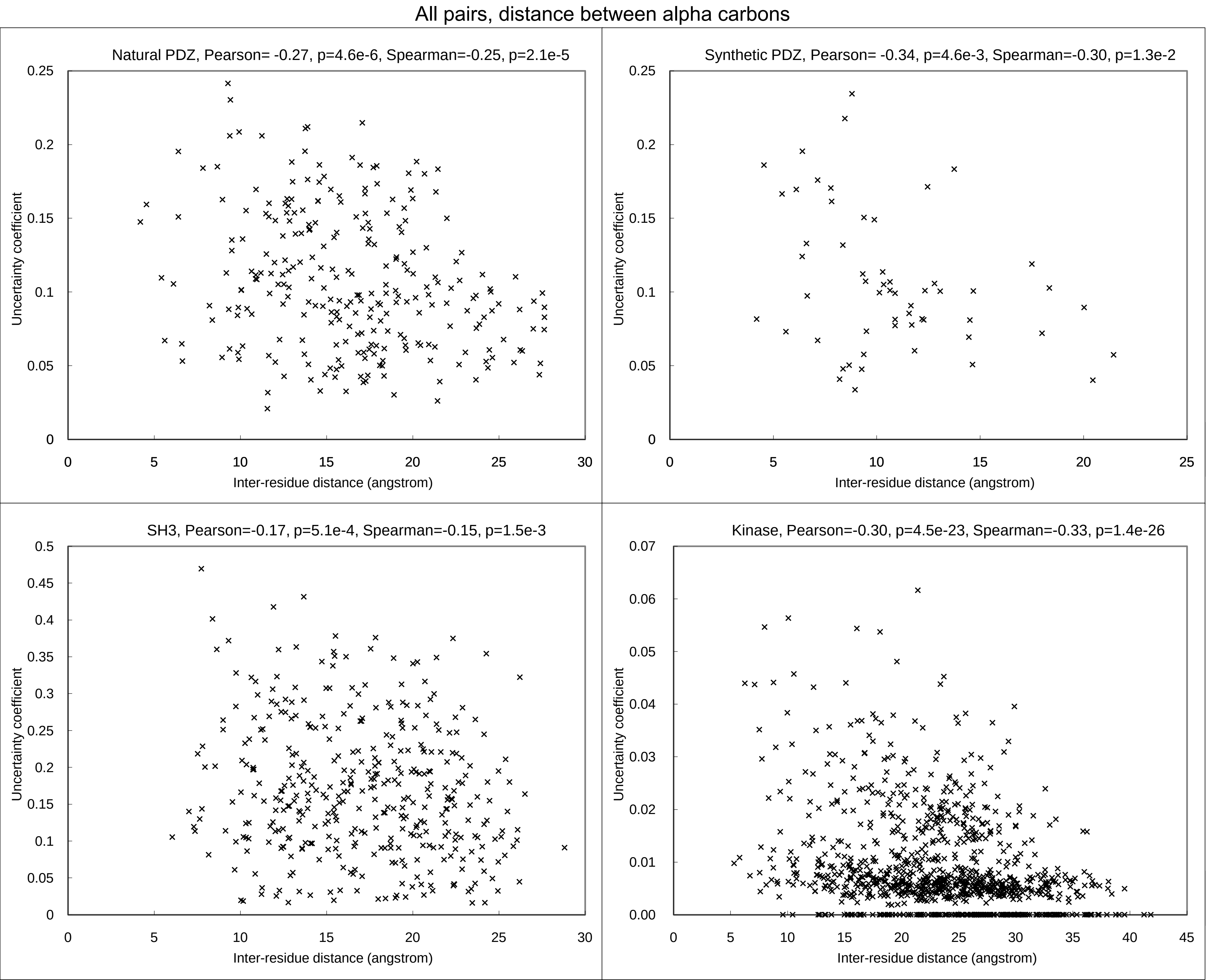

All pairs, distance between alpha carbons
Natural PDZ, Pearson= -0.27, p=4.6e-6, Spearman=-0.25, p=2.1e-5
Synthetic PDZ, Pearson= -0.34, p=4.6e-3, Spearman=-0.30, p=1.3e-2
SH3, Pearson=-0.17, p=5.1e-4, Spearman=-0.15, p=1.5e-3
Kinase, Pearson=-0.30, p=4.5e-23, Spearman=-0.33, p=1.4e-26
Uncertainty coefficient
Inter-residue distance (angstrom)
