# Supplementary figures and images for: Identification of specificity determining residues in peptide recognition domains using an information theoretic approach applied to large-scale binding maps
Source: BMC Biol. 2011 Aug 11;9:53. doi: 10.1186/1741-7007-9-53 (PMC3224579; doi:10.1186/1741-7007-9-53)

# All pairs, distance between closest atoms (minus sum of van der Waals' radii)

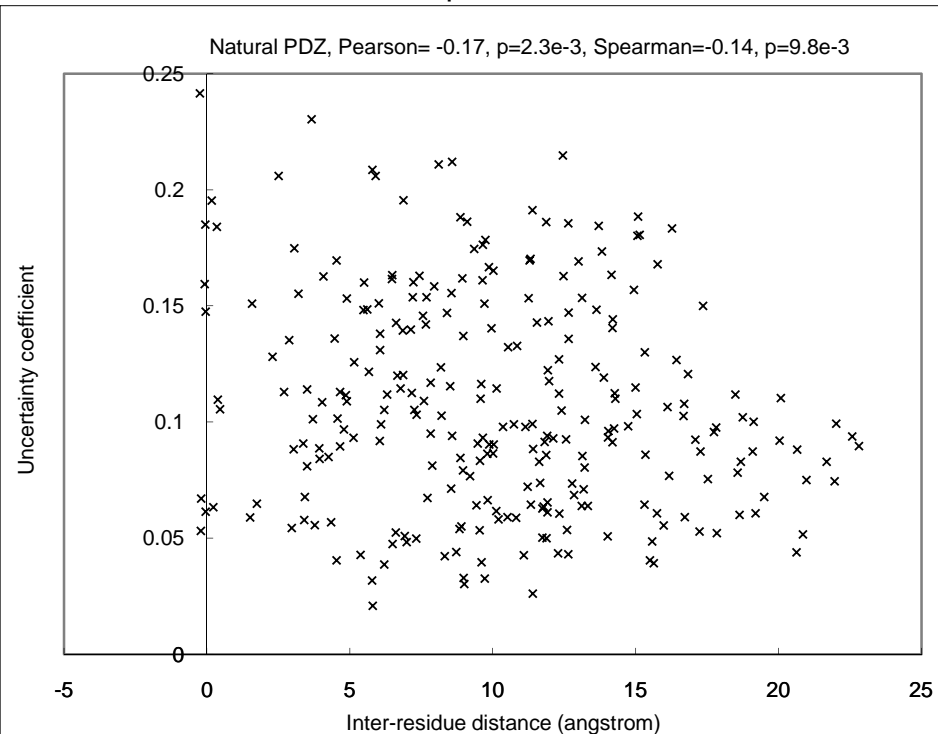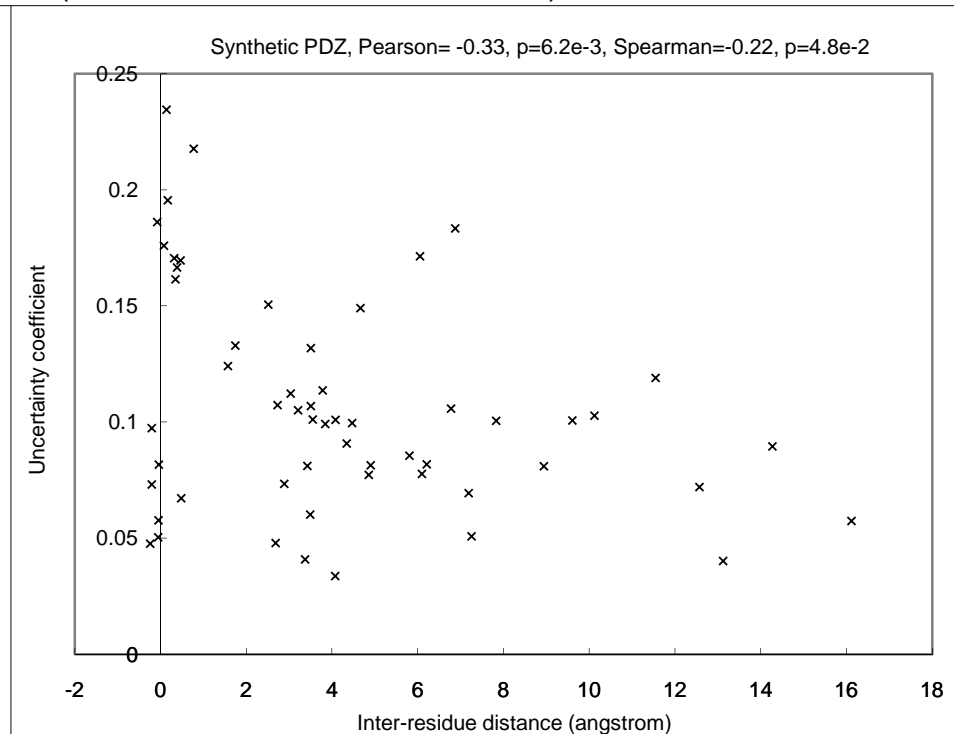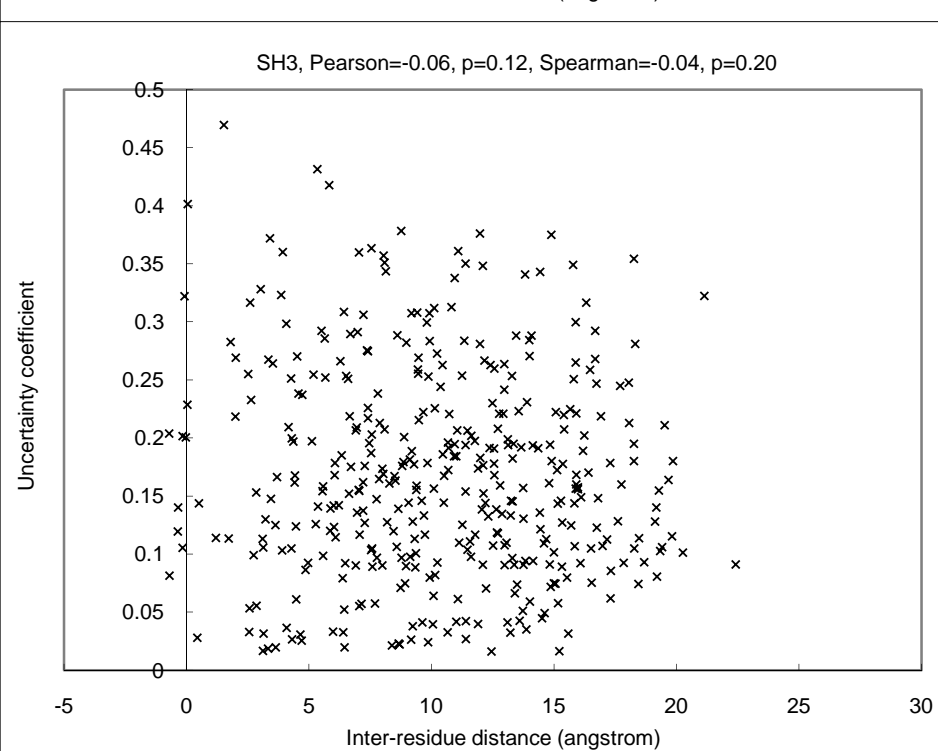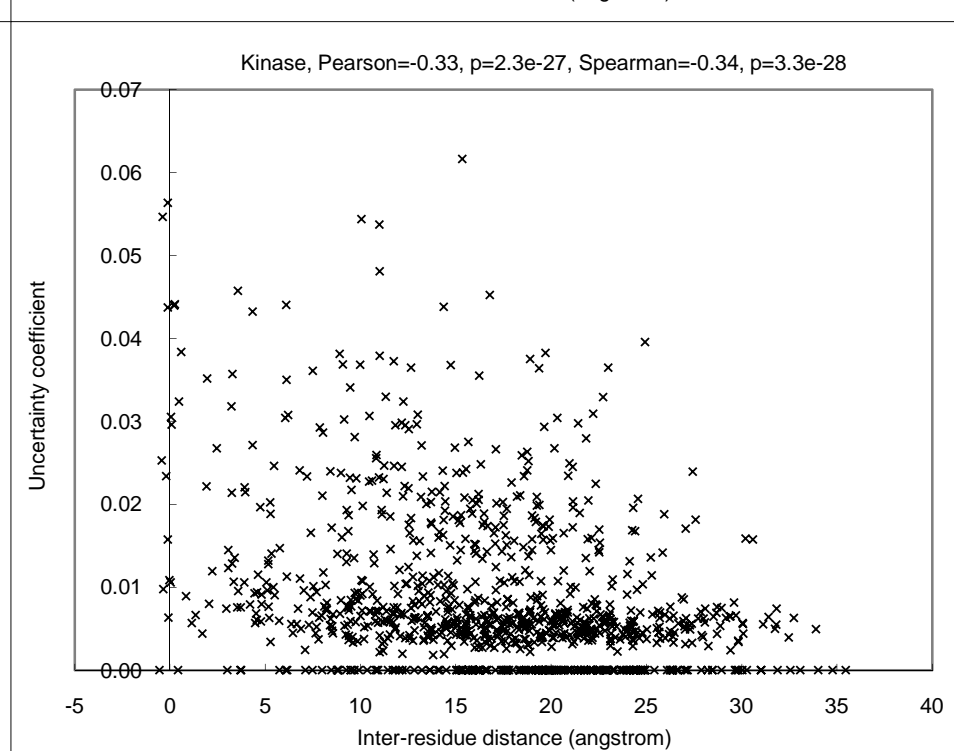

Supplement: Additional file 3 — Correlation between covariation score and physical proximity between each PRD site and each PWM position for the three types of PRDs when distances are computed between the closest atoms minus their van der Waal's radii. Figure S3 [file 1741-7007-9-53-S3.PDF]

# All pairs, distance between alpha carbons

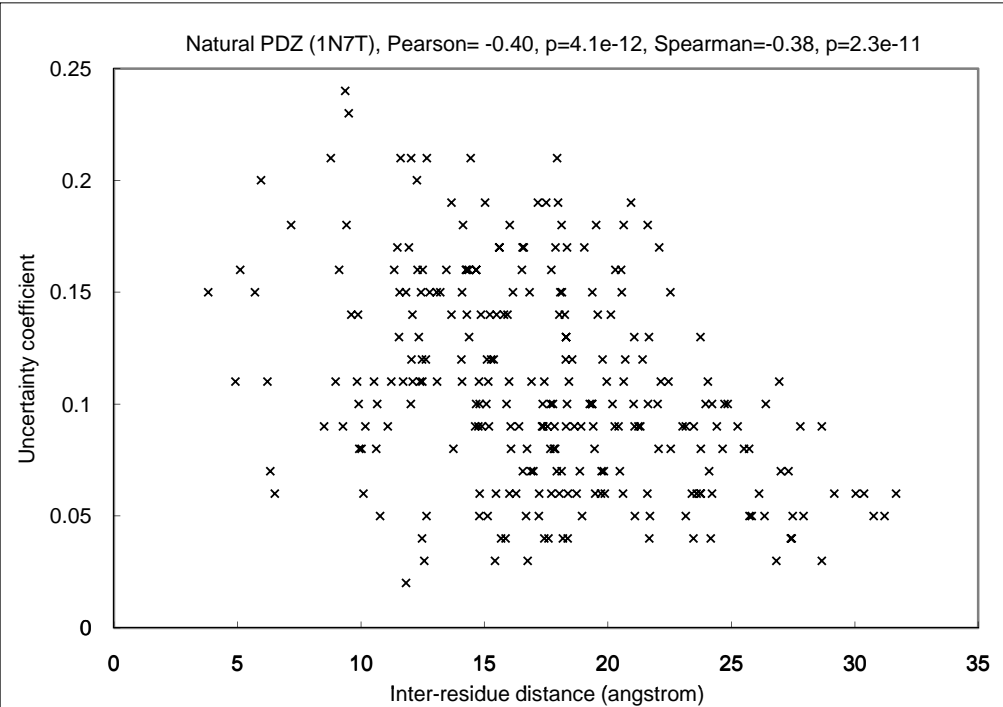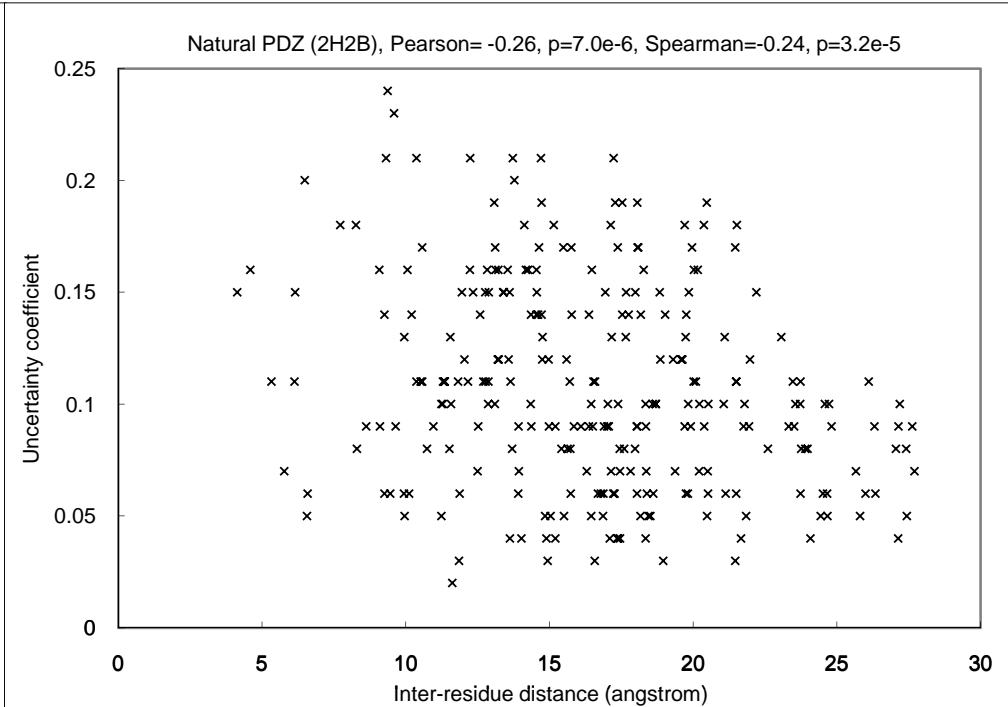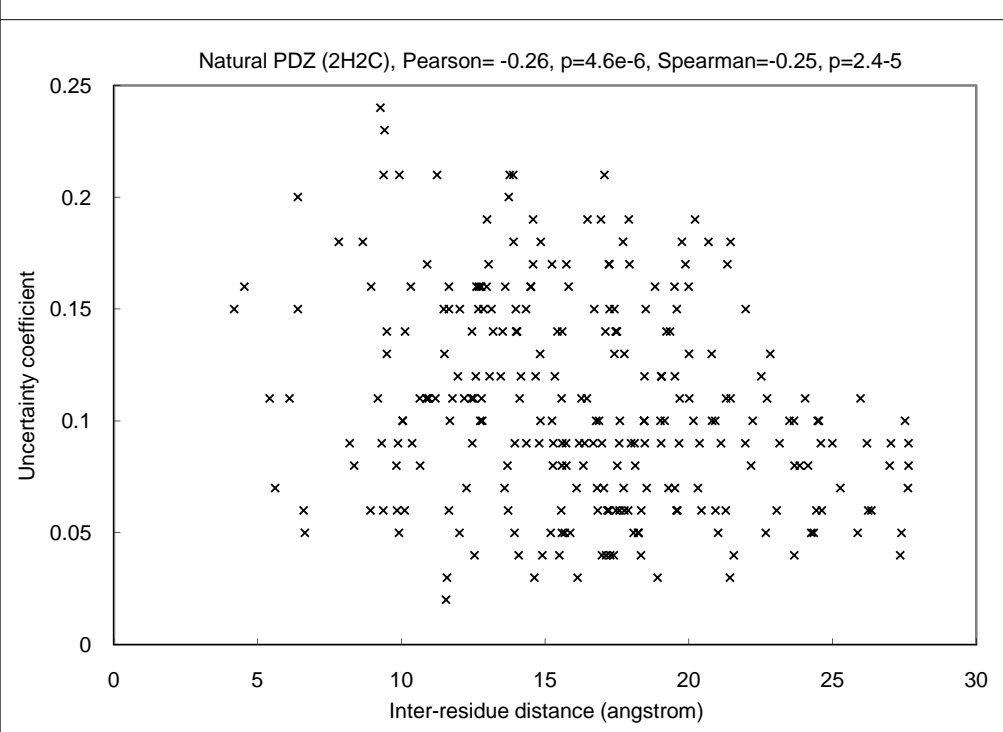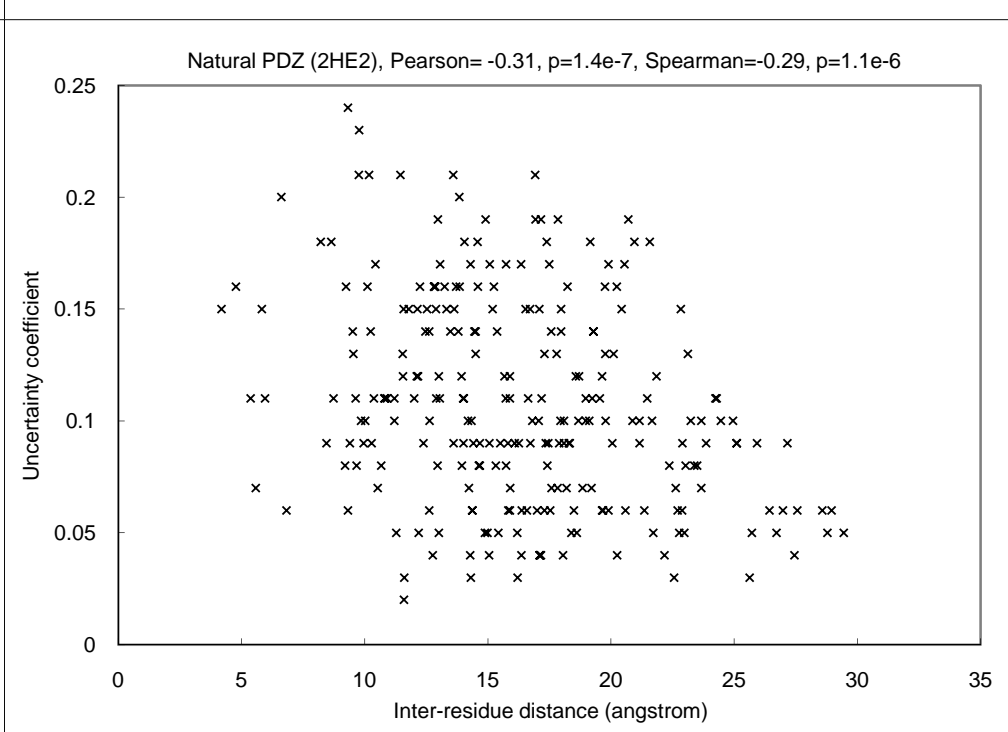

Supplement: Additional file 4 — Correlation between covariation score and physical proximity between each PRD site of a PDZ domain and each PWM position when distances are computed between alpha carbon atoms in four different PDB structures. Figure S4 [file 1741-7007-9-53-S4.PDF]

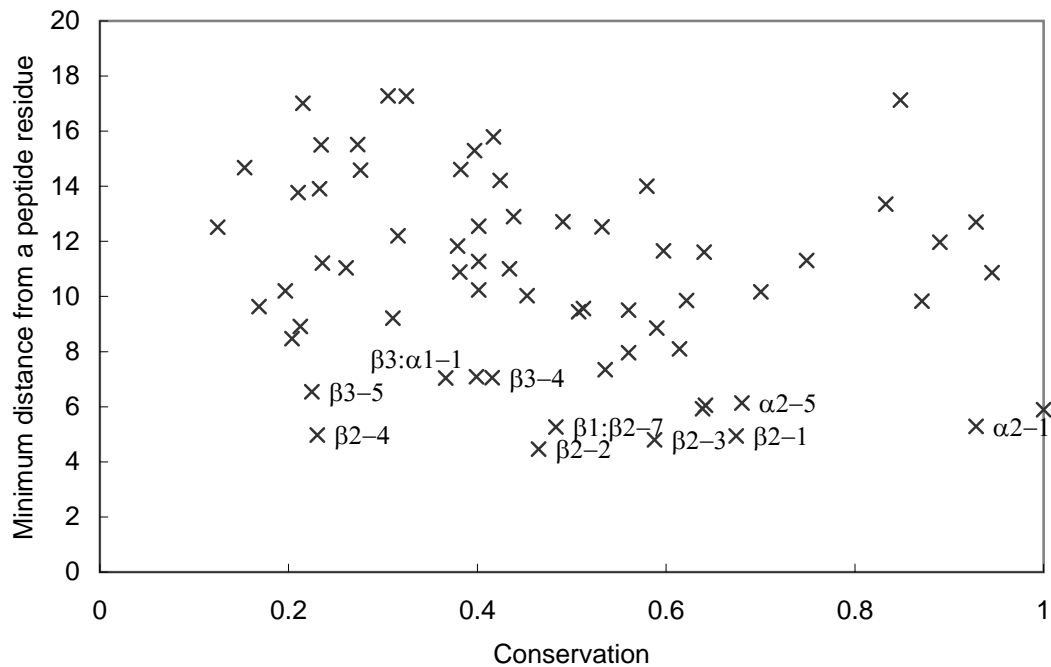

Supplement: Additional file 5 — Conservation and distance to the closest peptide residue of each PDZ domain site. For each site on the domain, we computed the total sequence weight of the sequences having a particular amino acid at the site. The conservation of the site is defined by the maximum of such total weights normalized by the total sequence weight of all sequences in the MSA. Figure S5. [file 1741-7007-9-53-S5.PDF]
